# Impact of ixazomib‐lenalidomide‐dexamethasone therapy on overall survival in multiple myeloma patients: Analysis of the emerging‐markets subgroup of the TOURMALINE‐MM1 trial

**DOI:** 10.1002/jha2.548

**Published:** 2022-08-31

**Authors:** Andrew Spencer, Olga Samoilova, Wee‐Joo Chng, Richard Labotka, Cong Li, Kwang‐Wei Wu, Nakul Saxena, Xu Yan, Jae Hoon Lee, Meral Beksac

**Affiliations:** ^1^ The Alfred Hospital‐Monash University Melbourne Victoria Australia; ^2^ State Budget Institution of Healthcare of Nizhny Novgorod Region Nizhny Novgorod Regional Clinical Hospital Nizhny Novgorod Russia; ^3^ National University Hospital Singapore Singapore; ^4^ Takeda Development Center Americas Lexington Massachusetts USA; ^5^ Takeda Pharmaceuticals International AG Singapore Singapore; ^6^ State Key Laboratory of Experimental Hematology National Clinical Research Center for Blood Diseases Haihe Laboratory of Cell Ecosystem Institute of Hematology and Blood Diseases Hospital Chinese Academy of Medical Sciences and Peking Union Medical College Tianjin China; ^7^ Gachon University Gil Medical Center Namdong‐gu South Korea; ^8^ Department of Hematology Ankara University School of Medicine Dikimevi Turkey

**Keywords:** emerging‐markets, ixazomib, multiple myeloma, overall survival, proteasome inhibitor

## Abstract

Ixazomib‐lenalidomide‐dexamethasone (ixazomib‐Rd) showed clinical efficacy over placebo‐Rd in patients with relapsed/refractory multiple myeloma (MM) in the TOURMALINE‐MM1 trial. Over a median follow‐up of ∼85 months, as patients showed disease progression, they received subsequent novel therapies that confounded the overall survival (OS) benefit. Here, we conducted a post hoc analysis in 148 patients from seven countries defined as emerging markets, with limited access to novel therapies for MM during the trial period, to describe the impact of these therapies on OS. Patients were randomised to ixazomib‐Rd (*n* = 71) or placebo‐Rd (*n* = 77). The median progression‐free survival (PFS) was 18.7 versus 10.2 months, with ixazomib‐Rd versus placebo‐Rd (hazard ratio [HR], 0.504; *p* = 0.008) demonstrating a statistically significant improvement as observed in the primary trial. The median OS improved by 32.6 months with ixazomib‐Rd over placebo‐Rd (63.5 vs. 30.9 months; HR, 0.794; *p* = 0.261); however, the statistically significant benefit seen in PFS was not observed for OS. Improvement with ixazomib‐Rd over placebo‐Rd was observed in overall response (81.7% vs. 64.9%; odds ratio [OR], 2.38; *p* = 0.019) and complete response (22.5% vs. 3.9%; OR, 7.57; *p* < 0.001). Patient‐reported quality of life and use of subsequent therapies were similar across treatment groups. No new safety concerns were identified. Compared with the main cohort, median OS was 10 months longer with ixazomib‐Rd and 21 months shorter with placebo‐Rd in this subgroup, indicating a clinically meaningful survival benefit of ixazomib‐Rd treatment in this patient population with limited access to subsequent novel therapies.

## 1 Introduction

Multiple myeloma (MM) is a cancer of the plasma cells characterised by anaemia, bone destruction, fatigue, hypercalcaemia and renal failure [1]. In 2020, more than 150,000 new cases of MM were diagnosed worldwide, and more than 100,000 deaths were recorded [2]. Australia and New Zealand are amongst countries with the highest age‐standardised incidence rate (ASIR) of MM in the world at 5 and 4.4 per 100,000 persons, respectively, while China has a low ASIR at 0.9 per 100,000 persons [3]. Although patients with newly diagnosed MM respond well to therapy, a large proportion become refractory during therapy or relapse afterwards.

Novel agents have expanded the therapeutic armamentarium for MM, which includes proteasome inhibitors (PIs) such as bortezomib, carfilzomib and ixazomib; immunomodulatory drugs (IMiDs) such as lenalidomide, thalidomide and pomalidomide; monoclonal antibodies such as the anti‐CD38 antibodies daratumumab and isatuximab and the anti‐SLAMF7 (CD319) antibody elotuzumab; and chimeric antigen receptor (CAR) T‐cell therapies [1, 4]. Daratumumab and elotuzumab were approved in 2015 by the United States (US) Food and Drug Administration (FDA) [5, 6] and in 2016 by the European Medicines Agency (EMA) [7, 8], while isatuximab was approved by both the FDA and EMA in 2020 [9, 10]. The availability of these drugs was limited before 2020 in the Asia Pacific, the Middle East, Eastern Europe and Latin America.

Clinical trials have shown that PIs are effective for treating patients with newly diagnosed, high‐risk MM, and these agents are currently the established regimen for relapsed or refractory (RR) MM in combination with IMiDs and corticosteroids [4]. Ixazomib, the first oral PI, was approved by the FDA in 2015 in combination with lenalidomide‐dexamethasone (Rd) for patients with MM who have received at least one prior therapy [11]. The pivotal global phase III TOURMALINE‐MM1 trial demonstrated that ixazomib‐Rd led to a significant improvement in progression‐free survival (PFS) over placebo‐Rd (median, 20.6 vs. 14.7 months; hazard ratio [HR], 0.74; *p* = 0.01) [12]. However, the subsequent final overall survival (OS) analysis of the TOURMALINE‐MM1 trial showed no significant improvement with ixazomib‐Rd versus placebo‐Rd in the overall population (median, 53.6 vs. 51.6 months; HR, 0.939; *p* = 0.495). The OS benefit in the overall population was confounded by the use of subsequent therapy (≥70% of patients). Specifically, PIs and daratumumab were administered more frequently in the placebo‐Rd group due to earlier disease progression compared with that in the ixazomib‐Rd group. Moreover, greater OS benefit with ixazomib‐Rd over placebo‐Rd was observed in patients who did not receive subsequent therapy compared with those who did [13].

The China Continuation study, a distinct regional expansion of the TOURMALINE‐MM1 trial comprising 115 patients, showed a statistically significant improvement with ixazomib‐Rd compared with placebo‐Rd in both median PFS (6.7 vs. 4.0 months; HR 0.598; *p* = 0.035) and median OS (25.8 vs. 15.8 months; HR 0.419; *p* = 0.001) [14]. The median PFS and OS in patients in the China Continuation study were relatively shorter than those reported in the TOURMALINE‐MM1 trial. Notably, patients in China did not have access to a broad range of novel approved or investigational agents available in North America and Europe. This result suggested that in countries with limited availability of novel therapeutic options, the PFS benefit with ixazomib‐Rd translates into an OS benefit [14].

Therefore, we conducted this post hoc subgroup analysis to assess and describe the OS benefit of ixazomib‐Rd in patients from emerging‐markets where there was limited availability or accessibility of novel agents for MM treatment, specifically daratumumab, elotuzumab, isatuximab and CAR T‐cell therapies, during the TOURMALINE‐MM1 trial [13].

## 2 Materials and methods

### 2.1 Study design and data sources

TOURMALINE‐MM1 trial was a randomised, placebo‐controlled, double‐blind, multicentre, phase III trial (ClinicalTrials.gov identifier: NCT01564537) [12]. The enrolment period was 28 August 2012 to 27 May 2014, and the data cutoff for the final analysis was 28 September 2020 [13]. This post hoc subgroup analysis included patients from emerging markets (Australia, China, New Zealand, Russia, Singapore, South Korea and Turkey), where there was limited availability or access to novel therapeutic agents for MM during the TOURMALINE‐MM1 trial. Full details of the study design, eligibility criteria and patient disposition have been described earlier [12, 13]. Briefly, the trial included patients ≥18 years of age with relapsed, refractory or relapsed and refractory MM who had received 1–3 prior lines of therapy. Patients who were refractory to prior lenalidomide‐ or PI‐based therapy were excluded [13].

For the TOURMALINE‐MM1 global trial, patients were randomly assigned 1:1 to receive ixazomib‐Rd or placebo‐Rd using a centralised interactive voice response system (IVRS). The dosage and administration schedule have been described earlier [12]. Randomisation was stratified by the number of prior therapies (1 vs. 2 or 3), previous exposure to PIs (naïve vs. exposed) and International Staging System (ISS) disease stage (I or II vs. III) [13].

### 2.2 Outcomes and assessments

OS was defined as the time from the date of randomisation to the date of death or date last known to be alive. PFS was defined as the time from the date of randomisation to the date of first documentation of disease progression or all‐cause death, whichever occurred first. Both OS and PFS were analysed in the intent‐to‐treat (ITT) population and in pre‐specified subgroups defined by age, ISS, cytogenetic risk markers, number of prior therapies, exposure to PIs and IMiDs, refractoriness to last prior therapy and RR status.

Other outcomes included disease response, patient‐reported health‐related quality of life (HRQoL) and safety. Disease response was defined and assessed per the International Myeloma Working Group Uniform Response Criteria [15] for overall response rate (ORR), complete response (CR), very good partial response, duration of response (DOR) and time to disease progression (TTP). HRQoL was evaluated using European Organisation for Research and Treatment of Cancer Quality of Life Questionnaire (EORTC QLQ) Core 30 (C30) and myeloma‐specific (MY20) modules [16, 17].

Adverse events (AEs) were coded using the Medical Dictionary for Regulatory Activities (MedDRA) version 16.0. Drug‐related (any drug in the drug combination) treatment‐emergent AEs (TEAEs) were analysed in the safety population and summarised using the National Cancer Institute Common Terminology Criteria for Adverse Events version 4.03. Patients were continuously assessed for new primary malignancies from study treatment initiation until death or study termination. Exploratory outcomes included the classes of subsequent therapies received by both study groups at follow‐up every 12 weeks from disease progression.

### 2.3 Statistical analyses

The ITT population included all patients from emerging markets who were randomised. The safety population included all patients who received at least one dose of the study drug or placebo. Formal hypothesis testing was not conducted. OS and PFS were evaluated using a closed sequential testing procedure [13] and assessed using the Kaplan–Meier methodology. Treatment groups were compared using a stratified Cox model to estimate the HRs and 95% confidence intervals (CIs) with two‐sided, stratified log‐rank tests for *p* values. Response rates were compared using a stratified Cochran‐Mantel‐Haenszel test, and odds ratios (ORs) were estimated using a logistic regression model.

## 3 RESULTS

### 3.1 Demographics and baseline clinical characteristics

This subgroup analysis included 148 patients (median age 63 years; 55% male; 77% white) from seven countries (emerging markets) who received either ixazomib‐Rd (*n* = 71) or placebo‐Rd (*n* = 77). Patient characteristics were well balanced between treatment groups. High‐risk cytogenetic abnormalities occurred in 22% of patients in the ITT population and were similarly distributed in the two treatment groups. Overall, 41%, 43% and 17% of patients had received 1, 2 and 3 prior therapies, respectively; 68% of patients had received bortezomib, and 52% had received IMiDs (Table 1).

**TABLE 1:** Baseline patient characteristics (ITT population)

Characteristics	Ixazomib‐Rd *n* = 71	Placebo‐Rd *n* = 77	Overall *N* = 148
Age, years			
Median (range)	63.0 (44–91)	64.0 (30–84)	63.0 (30–91)
>65, *n* (%)	27 (38)	35 (45)	62 (42)
Male, *n* (%)	38 (54)	43 (56)	81 (55)
Race^a^, *n* (%)			
White	54 (76)	60 (78)	114 (77)
Asian	10 (14)	11 (14)	21 (14)
Others	6 (8)	2 (3)	8 (5)
Not reported	1 (1)	4 (5)	5 (3)
ECOG PS^b^, *n* (%)			
0	30 (42)	37 (48)	67 (45)
1	38 (54)	36 (47)	74 (50)
2	3 (4)	4 (5)	7 (5)
ISS disease stage at study entry^c^, n (%)			
I	48 (68)	49 (64)	97 (66)
II	17 (24)	19 (25)	36 (24)
III	6 (8)	9 (12)	15 (10)
Creatinine clearance, ml/min/1.73 m^2^, *n* (%)			
Median (range)	96.1 (31–233)	86.2 (27–233)	87.5 (27–233)
<30	0 (0)	1 (1)	1 (<1)
30 to <60	9 (13)	21 (27)	30 (20)
60 to <90	25 (35)	20 (26)	45 (30)
≥90	37 (52)	35 (45)	72 (49)
Time since initial diagnosis of MM, months, median (range)	45.2 (9–174)	42.2 (5–306)	43.1 (5–306)
Cytogenetic features^d^, *n* (%)			
Standard‐risk cytogenetic abnormalities	33 (54)	48 (71)	81 (63)
High‐risk cytogenetic abnormalities	14 (23)	14 (21)	28 (22)
Data not available	24 (39)	15 (22)	39 (30)
Number of prior therapies^e^, *n* (%)			
1	30 (42)	30 (39)	60 (41)
2	30 (42)	33 (43)	63 (43)
3	11 (15)	14 (18)	25 (17)
Prior stem cell transplantation, *n* (%)	34 (48)	34 (44)	68 (46)
Disease category^f^, *n* (%)			
Relapsed	47 (66)	48 (62)	95 (64)
Refractory	10 (14)	9 (12)	19 (13)
Relapsed and refractory	13 (18)	20 (26)	33 (22)
Prior PI therapy, *n* (%)	47 (66)	54 (70)	101 (68)
Bortezomib	47 (66)	54 (70)	101 (68)
Carfilzomib	0 (0)	0 (0)	0 (0)
Disease refractory to any prior PI therapy^g^	4 (9)	5 (9)	9 (9)
Prior IMiD therapy^h^, *n* (%)	33 (46)	44 (57)	77 (52)
Lenalidomide	2 (3)	2 (3)	4 (3)
Thalidomide	33 (46)	43 (56)	76 (51)
Disease refractory to any prior IMiD therapy	13 (39)	19 (43)	32 (42)

Abbreviations: ECOG PS, Eastern Cooperative Oncology Group performance status; IMiD, immunomodulatory drug; ISS, International Staging System; ITT, intent‐to‐treat; MM, multiple myeloma; PI, proteasome inhibitor; Rd, lenalidomide and dexamethasone.

^a^ Race was self‐reported.

^b^ ECOG PS is scored on a scale from 0 to 5, with 0 indicating no symptoms and higher scores indicating increasing disability related to tumour.

^c^ ISS consists of three stages: stage I, serum β2‐microglobulin level <3.5 mg/L (300 nmol/L) and albumin level ≥3.5 g/dl; stage II, neither stage I nor III; and stage III, serum β2‐microglobulin level ≥5.5 mg/L (470 nmol/L). Higher stages indicate more severe disease.

^d^ High‐risk cytogenetic abnormalities were detected by fluorescence in situ hybridisation analysis and were defined as chromosome 17p deletion [del(17p)], translocation between chromosomes 4 and 14 [t(4;14)] and translocation between chromosomes 14 and 16 [t(14;16)]. A total of six patients in the ixazomib‐Rd group and seven patients in the placebo‐Rd group had del(17p) alone; eight and six patients had t(4;14) alone; and zero and one patient had t(14;16) alone, respectively. Standard‐risk cytogenetic abnormalities were defined as the absence of high‐risk abnormalities in the samples that were available for evaluation; samples from some patients were not available for testing because these were missing or clotted or due to other reasons. Percentages are based on the number of patients with sample collected for magnetic resonance imaging/computed tomography.

^e^ The number of prior therapies was determined by the sponsor in a blinded medical review of data on prior therapy.

^f^ The relapsed category comprised patients who relapsed from ≥1 previous treatment but were not refractory to any previous treatment. The refractory category comprised patients who were refractory to ≥1 previous treatment but did not relapse from any previous treatment. The relapsed and refractory category comprised patients who relapsed from ≥1 previous treatment and additionally were refractory to ≥1 previous treatment. For this study, refractory disease was defined as disease progression on treatment or progression within 60 days after the last dose of a given therapy.

^g^ Refractoriness to any prior PI therapy was determined by the sponsor in a blinded medical review, and percentages are based on the number of patients who received PI therapy.

^h^ Percentages are based on the number of patients exposed to prior IMiDs in each group.

### 3.2 Efficacy

**FIGURE 1:**
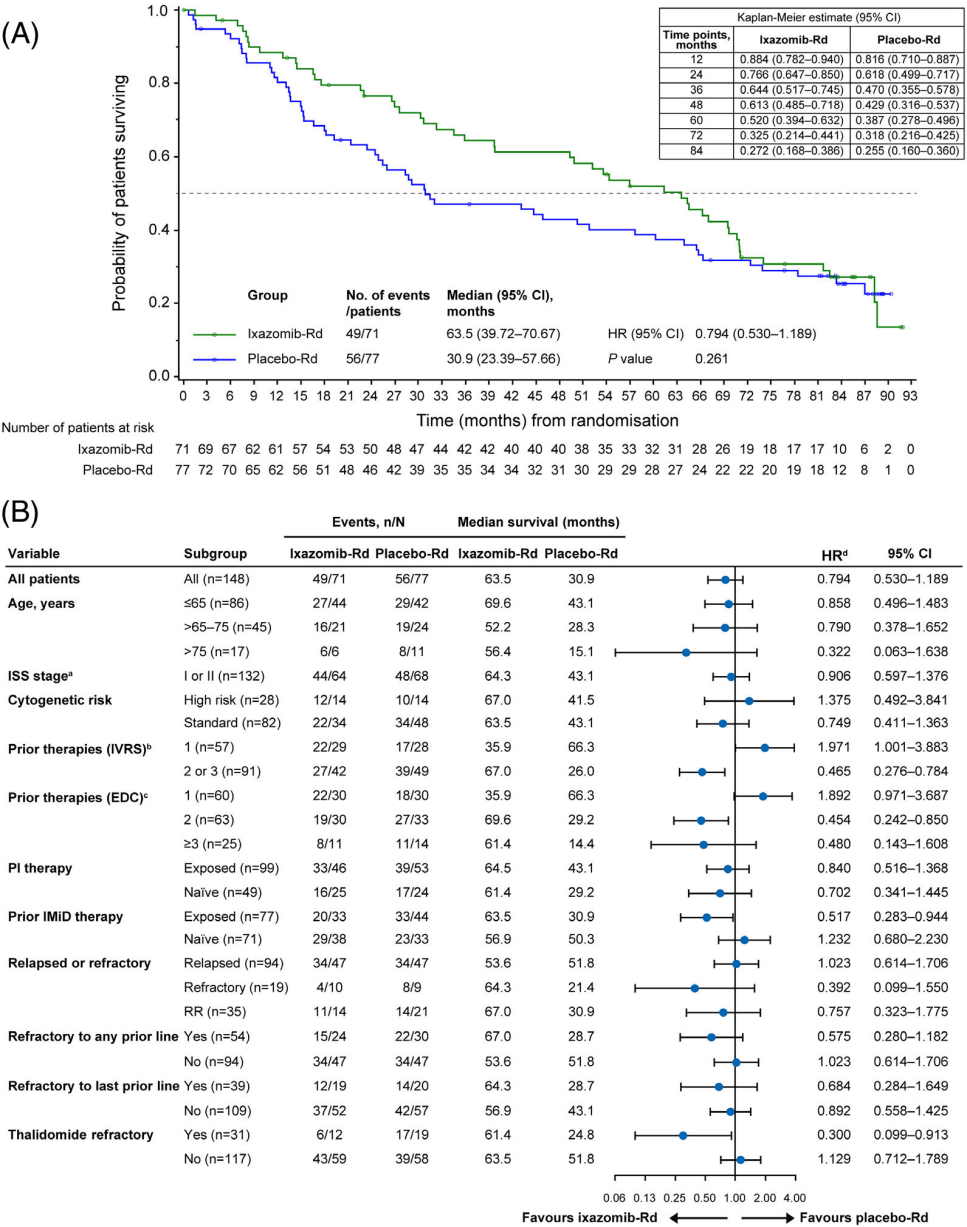
Kaplan–Meier analysis of overall survival (OS) in (A) the ITT population and (B) by patient subgroups. CI, confidence interval; EDC, electronic data capture; HR, hazard ratio; IMiD, immunomodulatory drug; ISS, International Staging System; ITT, intent‐to‐treat; IVRS, interactive voice response system; n, number in subgroup; N, number in study group; OS, overall survival; PI, proteasome inhibitor; Rd, lenalidomide and dexamethasone; RR, relapsed and refractory. ^a^ISS stage stratification per IVRS at enrolment. ^b^Prior therapy stratification per IVRS at enrolment. ^c^Prior therapies as determined by Takeda medical review of prior therapy data. ^d^HR based on stratified Cox proportional hazard regression model.

At data cutoff, median follow‐up was 85.4 months in the ixazomib‐Rd group and 84.5 months in the placebo‐Rd group, with 49 and 56 deaths, respectively. Median OS was 32.6 months longer in the ixazomib‐Rd group (63.5 months) versus the placebo‐Rd group (30.9 months; HR, 0.794; 95% CI, 0.530–1.189; *p* = 0.261) (Figure 1A). The survival probability (ixazomib‐Rd vs. placebo‐Rd) at different time points showed a lower survival rate in the placebo‐Rd group from 36 to 60 months: 76.6% versus 61.8% at 24 months, 64.4% versus 47.0% at 36 months, 61.3% versus 42.9% at 48 months and 52.0% versus 38.7% at 60 months (Figure 1A). A significantly lower risk of death with ixazomib‐Rd versus placebo‐Rd was observed in pre‐specified subgroups of patients with ≥2 prior therapies by IVRS (HR, 0.465; 95% CI, 0.276–0.784), with two prior therapies by electronic data capture (EDC) (HR, 0.454; 95% CI, 0.242–0.850), with prior exposure to IMiDs (HR, 0.517; 95% CI, 0.283–0.944) and refractory to thalidomide (HR, 0.3; 95% CI, 0.099–0.913) (Figure 1B). Additionally, a trend towards lower risk of death with ixazomib‐Rd versus placebo‐Rd was observed in patients >75 years of age, with ≥3 prior therapies by EDC and refractory disease.

**FIGURE 2:**
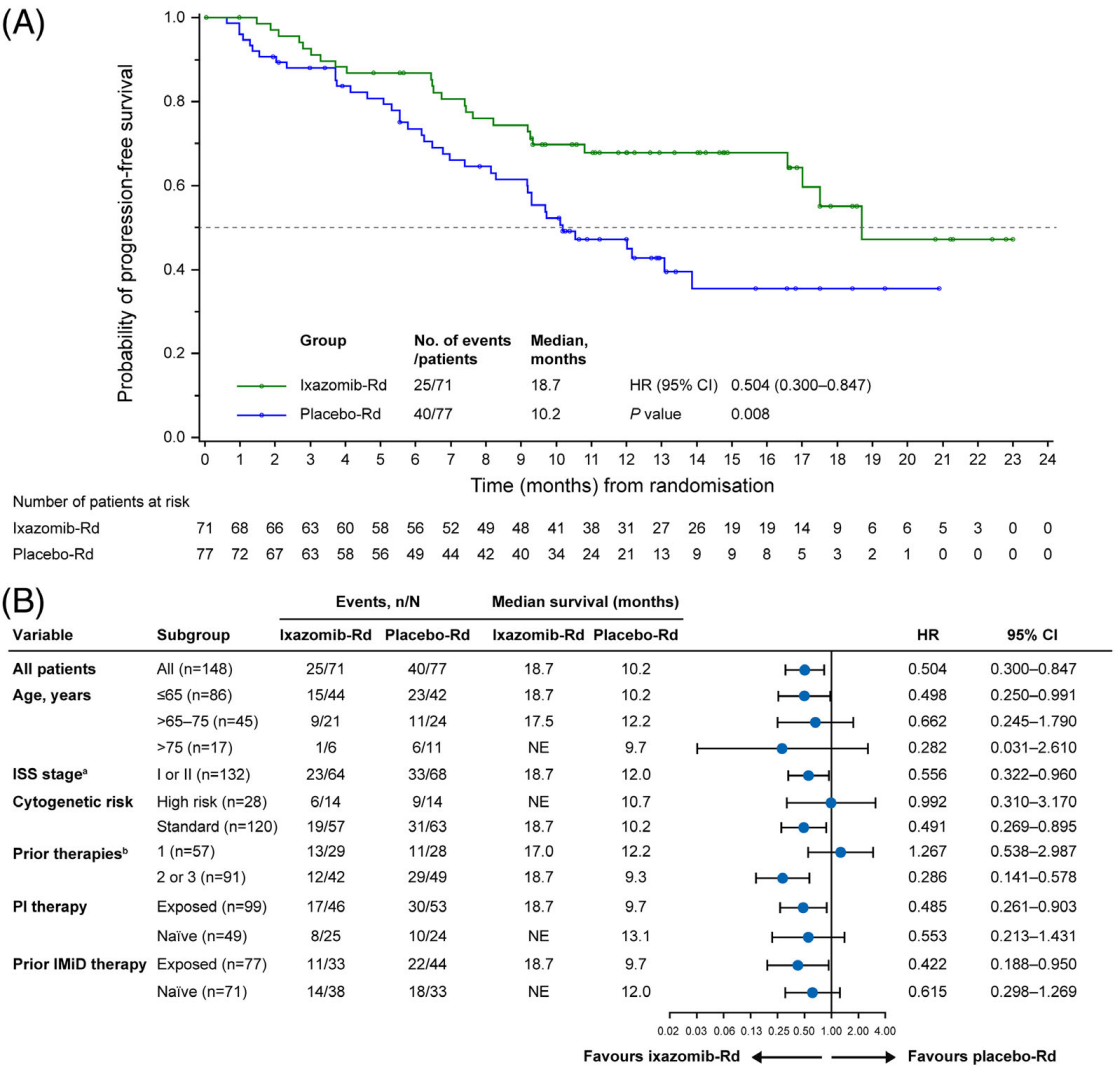
Kaplan**–**Meier analysis of PFS in (A) the ITT population and (B) by patient subgroups. CI, confidence interval; HR, hazard ratio; IMiD, immunomodulatory drug; ISS, International Staging System; ITT, intent‐to‐treat; *n*, number in subgroup; *N*, number in study group; NE, not estimable; PFS, progression‐free survival; PI, proteasome inhibitor; Rd, lenalidomide and dexamethasone. ^a^ISS stage stratification per IVRS at enrolment. ^b^Prior therapy stratification per IVRS at enrolment.

Median PFS was significantly longer in the ixazomib‐Rd group versus the placebo‐Rd group (18.7 vs. 10.2 months; HR, 0.504; 95% CI, 0.300–0.847; *p* = 0.008) (Figure 2A). A lower risk of disease progression with ixazomib‐Rd versus placebo‐Rd was seen in most of the pre‐specified subgroups, including patients ≤65 years of age (HR, 0.498; 95% CI, 0.250–0.991), with ISS stage I or II (HR, 0.556; 95% CI, 0.322–0.960), standard‐risk cytogenetics (HR, 0.491; 95% CI, 0.269–0.895), ≥2 prior therapies (HR, 0.286; 95% CI, 0.141–0.578), IMiD exposure (HR, 0.422; 95% CI, 0.188–0.95) and PI exposure (HR, 0.485; 95% CI, 0.261–0.903) (Figure 2B). All other subgroups except patients with one prior therapy demonstrated a trend towards lower risk of disease progression favouring the ixazomib‐Rd group.

The ORR was significantly higher in the ixazomib‐Rd group (81.7%) versus the placebo‐Rd group (64.9%; OR, 2.38; 95% CI, 1.10–5.13; *p* = 0.019). Similarly, CR was observed in a significantly higher proportion of patients in the ixazomib‐Rd group (22.5%) versus the placebo‐Rd group (3.9%; OR, 7.57; 95% CI, 2.06–27.80; *p* < 0.001). The median TTP was 18.7 and 12.0 months in the ixazomib‐Rd and placebo‐Rd groups, respectively (HR, 0.528; 95% CI, 0.305–0.913; *p* = 0.020) (Table 2).

**TABLE 2:** Best responses^a^ to study regimen and time to progression (ITT population)

Response	Ixazomib‐Rd *n* = 71	Placebo‐Rd *n* = 77	*p*‐value
ORR^b^			
Patients with response, *n* (%)	58 (81.7)	50 (64.9)	0.019
Response rate, 95% CI (%)	70.7–89.9	53.2–75.5	
≥VGPR^c^			
Patients with response, *n* (%)	39 (54.9)	23 (29.9)	0.002
Response rate, 95% CI (%)	42.7–66.8	20.0–41.4	
Best response			
CR^d^			
Patients with response, *n* (%)	16 (22.5)	3 (3.9)	< 0.001
Response rate, 95% CI (%)	13.5–34.0	0.8–11.0	
Stringent CR^e^			
Patients with response, *n* (%)	5 (7.0)	1 (1.3)	
Response rate, 95% CI (%)	2.3–15.7	0.0–7.0	
PR^f^			
Patients with response, *n* (%)	42 (59.2)	47 (61.0)	
Response rate, 95% CI (%)	46.8–70.7	49.2–72.0	
VGPR			
Patients with response, *n* (%)	23 (32.4)	20 (26.0)	
Response rate, 95% CI (%)	21.8–44.5	16.6–37.2	
Stable disease^g^			
Patients with response, *n* (%)	6 (8.5)	17 (22.1)	
Response rate, 95% CI (%)	3.2–17.5	13.4–33.0	
Median time to response, months	1.1	1.9	0.020
Median DOR (≥PR)^h^, months	NE	9.3	
Median TTP^i^, months	18.7	12.0	0.020

Abbreviations: CI, confidence interval; CR, complete response; DOR, duration of response; ITT, intent‐to‐treat; NE, not estimable; ORR, overall response rate; PR, partial response, Rd, lenalidomide and dexamethasone; TTP, time to disease progression; VGPR, very good partial response.

^a^ Best response after X cycles represents the best confirmed or unconfirmed response within X cycles for all ITT patients. The timeframe is from cycle 1 day 1 (C1D1) to cycle X C(X+1)D1 or end of treatment, whichever comes earlier. Percentages are based on the total number of patients in the ITT population who reached the C(X+1)D1 visit or discontinued.

^b^ ORR is defined as the proportion of ITT patients who achieved PR or better, estimated as ORR = CR+VGPR+PR.

^c^ VGPR is a subset of PR. VGPR is defined as serum and urine M‐protein being detectable by immunofixation but not on electrophoresis or ≥90% reduction in serum M‐protein plus urine M‐protein level of <100 mg per 24 h.

^d^ CR is defined as no detection of M‐protein in the serum and urine, disappearance of soft‐tissue plasmacytomas and <5% of plasma cells in the bone marrow.

^e^ Stringent CR is a subset of CR. Criteria for a stringent CR include the criteria for a CR plus a normal free light chain ratio and absence of clonal plasma cells, as assessed using immunohistochemical analysis or immunofluorescence.

^f^ PR is defined as reduction of serum M‐protein by ≥50% and urine M‐protein by ≥90% (<200 mg per 24 h) and ≥50% reduction in the size of soft‐tissue plasmacytomas.

^g^ Stable disease is defined as the response not meeting any of the criteria for CR, PR, VGPR or progressive disease.

^h^ DOR was measured as the time from the date of first documentation of PR or better to the date of first documented progression. The number of patients for this analysis in the ixazomib‐Rd and placebo‐Rd groups was 58 and 49, respectively.

^i^ TTP was estimated as the time from randomisation to the date of first documented progression.

The EORTC QLQ‐C30 global health status/QoL subscale scores and the EORTC QLQ‐MY20 scores for disease symptoms and side effects of treatment subscales were similar between the ixazomib‐Rd and placebo‐Rd groups over the course of the follow‐up (Figure S1).

### 3.3 Safety

The safety population included 70 patients in the ixazomib‐Rd group and 77 in the placebo‐Rd group. One patient in the ixazomib‐Rd group received the study drug but did not complete the treatment and was removed from the safety population. The treatment duration was similar for the ixazomib‐Rd and placebo‐Rd groups: a median of 13 and 12 cycles of treatment with a median exposure of 361 and 327 days, respectively (Table S1). The rates of drug‐related serious AEs, discontinuation of the study regimen because of AEs and death during the treatment period were similar between the ixazomib‐Rd and placebo‐Rd groups (Table 3). The incidence of grade ≥3 drug‐related AEs was 61% in the ixazomib‐Rd group versus 45% in the placebo‐Rd group (Table 3). Grade ≥3 AEs occurring with a ≥5% higher incidence in the ixazomib‐Rd group versus the placebo‐Rd group were diarrhoea (10% vs. 0%) insomnia (7.1% vs. 0.0%) and thrombocytopenia (12.9% vs. 7.8%) (Table S2). Primary malignancy was observed in 12 patients (16.9%) in the ixazomib‐Rd group and 10 patients (13.0%) in the placebo‐Rd group.

**TABLE 3:** Summary of TEAEs (safety population)

Category, *n* (%)	Ixazomib‐Rd *n* = 70	Placebo‐Rd *n* = 77
Any AE	68 (97)	77 (100)
Grade ≥3 AE	53 (76)	58 (75)
Drug‐related AE	64 (91)	71 (92)
Grade ≥3 drug‐related AE	43 (61)	35 (45)
SAE	37 (53)	49 (64)
Drug‐related SAE	17 (24)	20 (26)
AEs resulting in study drug dose reduction	44 (63)	31 (40)
AEs resulting in study drug dose modification^a^	54 (77)	48 (62)
AEs resulting in any study drug discontinuation	23 (33)	21 (27)
AEs resulting in all study drug discontinuation	15 (21)	16 (21)
On‐study deaths^b^	5 (7)	8 (10)

Abbreviations: AE, adverse event; Rd, lenalidomide and dexamethasone; SAE, serious adverse event; TEAE, treatment‐emergent adverse event.

^a^ Dose modification includes dose reduction, dose increase, dose delay and dose discontinuation, which may be in relation to any of the three study drugs.

^b^ On‐study deaths are defined as deaths that occur within 30 days of the last dose of the study drug.

### 3.4 Subsequent therapy

In both ixazomib‐Rd and placebo‐Rd groups, a similar proportion of patients (64% vs. 65%, respectively) received subsequent anticancer therapy following study treatment completion. Overall, similar proportions of patients in the ixazomib‐Rd and placebo‐Rd groups were administered corticosteroids (61% vs. 64%), IMiDs (43% vs. 44%), alkylating agents (44% vs. 42%) and PIs (36% vs. 42%) (Table 4).

**TABLE 4:** Subsequent therapies (safety population)

Antineoplastic therapy reported, *n* (%)	Ixazomib‐Rd *n* = 70	Placebo‐Rd *n* = 77
Patients with ≥1 subsequent anticancer therapy	45 (64)	50 (65)
Corticosteroids^a^	43 (61)	49 (64)
Alkylating agents^b^	31 (44)	32 (42)
IMiDs^c^	30 (43)	34 (44)
PIs^d^	25 (36)	32 (42)
Daratumumab	3 (4)	9 (12)
Elotuzumab	0 (0)	2 (3)
Other monoclonal antibodies^e^	2 (3)	0 (0)
Anthracyclines	6 (9)	4 (5)
Topoisomerase inhibitors	4 (6)	3 (4)
Vinca alkaloids	2 (3)	0 (0)
Antimetabolites	0 (0)	2 (3)

Abbreviations: IMiD, immunomodulatory drug; PI, proteasome inhibitor; Rd, lenalidomide and dexamethasone.

^a^ This included dexamethasone, prednisone, prednisolone, methylprednisolone and hydrocortisone. Dexamethasone was the most administered corticosteroid at 57%, overall.

^b^ Alkylating agents included cyclophosphamide, melphalan, bendamustine, cisplatin, lomustine and procarbazine; cyclophosphamide was administered to the majority (35%), overall.

^c^ Overall, 12% and 13% of patients received lenalidomide and thalidomide, respectively, but 26% received pomalidomide.

^d^ Bortezomib was administered to the majority (29%), followed by carfilzomib (14%) and ixazomib (2%) in the overall subgroup.

^e^ This category includes isatuximab.

## 4 Discussion

This post hoc subgroup analysis of the TOURMALINE‐MM1 trial aimed to evaluate survival benefit with ixazomib‐Rd over placebo‐Rd in patients from emerging‐market countries where novel agents for MM therapy were not accessible at the time of the study. This setting is similar to that of the China Continuation study, in which novel agents for MM therapy were not available during the study period, and the PFS benefit of ixazomib‐Rd over placebo‐Rd was translated to significant OS benefit [14]. Similarly, in this subgroup analysis, we demonstrated a trend of improved survival with ixazomib‐Rd compared with placebo‐Rd.

A statistically significant improvement in PFS was observed in the emerging‐markets subgroup, similar to the main cohort. In this subgroup, the median OS with ixazomib‐Rd was ∼33 months longer than that with placebo‐Rd (63.5 vs. 30.9 months). This is in contrast to the main trial, which showed a similar median OS between the two groups (53.6 vs. 51.6 months) [13]. Although the difference in OS in this subgroup analysis was not statistically significant, it shows a meaningful clinical benefit for ixazomib‐Rd over placebo‐Rd. Notably, lower survival probability in the placebo‐Rd group versus ixazomib‐Rd group was apparent from 36 to 60 months, but this trend was not observed at longer follow‐up periods ≥72 months. The variability in results may be reflective of the small sample size at ≥72 months and potentially increased access to novel agents that were being approved in certain emerging markets. For example, elotuzumab, daratumumab and isatuximab were approved in Australia in 2016 [18], 2017 [19] and 2020 [20], respectively, while daratumumab was approved in China in 2019 [21]. However, a lower proportion of patients treated with ixazomib‐Rd versus placebo‐Rd received subsequent therapies of daratumumab (4% vs. 12%) and elotuzumab (0% vs. 3%), and the overall use of novel subsequent therapies remained low.

This post hoc analysis demonstrated a greater OS benefit with ixazomib‐Rd over placebo‐Rd among patients with adverse prognostic factors such as ≥2 prior therapies, prior exposure to IMiDs and refractory to prior therapies such as thalidomide. Additionally, a trend towards lower risk of death was observed with ixazomib‐Rd versus placebo‐Rd in patients >75 years of age, with ≥3 prior therapies by EDC and refractory disease. Considering these subgroups represent patients with poor prognosis, this analysis indicated that treatment with ixazomib‐Rd is likely to provide considerable survival benefit in such patients. Further studies should be conducted to confirm these potential predictors for efficacy. In this study, compared with the placebo‐Rd group, the ixazomib‐Rd group had a higher ORR, and more patients treated with ixazomib‐Rd achieved CR. Compared to the main cohort, the emerging‐markets subgroup of ixazomib‐Rd–treated patients had a higher ORR (78.0% vs. 81.7%) and CR (12.0% vs. 22.5%). The DOR with ixazomib‐Rd was not estimable in the subgroup, whereas it was 20.5 months in the main cohort [12]. No new safety concerns for the use of ixazomib‐Rd in patients with RRMM were identified in this subgroup analysis.

When compared with the main cohort, the median OS in the emerging‐markets subgroup was approximately 10 months longer in the ixazomib‐Rd group and almost 21 months shorter in the placebo group. Most attributes of the emerging‐market subgroup population were similar to those of the main cohort, with a few key differences. Specifically, this subgroup had a higher proportion of patients who underwent >1 prior therapy (59% vs. 39%) and were refractory to prior IMiDs (42% vs. 23%). In addition, fewer patients had received stem cell transplantation (46% vs. 57%) or had prior exposure to lenalidomide (3% vs. 12%). Although the proportion of patients who received prior PI therapy was similar in the emerging‐markets subgroup and in the main cohort (68% vs. 70%, respectively), the proportion of patients refractory to PIs was higher in the emerging‐markets subgroup (9% vs. 2%). A similar proportion of patients in the emerging‐markets subgroup and the main cohort received subsequent therapies (65% vs. 71%, respectively); however, as would be expected, the emerging‐markets subgroup reported lower use of novel therapies versus the main cohort (daratumumab [8% vs. 21%], elotuzumab [1% vs. 4%] and isatuximab [1% vs. 3%]). In the main cohort, 80% of patients were from North America and Europe and had access to novel therapeutic agents for subsequent therapy, which may have confounded the interpretation of OS [13]. Conversely, in the current study, the effect of novel therapeutics is likely to be less pronounced. Compared with the main cohort, patients in emerging‐market countries reported here may have a poorer prognosis and may be more likely to experience a considerable benefit in OS with ixazomib‐Rd treatment, especially considering the low rates of novel therapy use. Taken together, the clinical characteristics and the minimal use of novel subsequent therapies in the emerging‐markets subgroup may explain the greater difference in survival probabilities between ixazomib‐Rd and placebo‐Rd treatments compared with the main cohort.

Triplet regimens containing a PI have demonstrated survival benefits in both clinical and real‐world studies. A combination of carfilzomib, with Rd, was evaluated for clinical efficacy in patients with RRMM who had received 1–3 prior therapies, in the ASPIRE trial [22]. Carfilzomib‐Rd significantly reduced the risk of death, with a median OS of 48.3 months compared with 40.4 months for placebo‐Rd (*p* = 0.045) [23]. In fact, clinical effectiveness of ixazomib‐Rd has been demonstrated in a real‐world, head‐to‐head, comparative study in patients with RRMM from the Czech Registry of Monoclonal Gammopathies. The median PFS was significantly improved with ixazomib‐Rd versus placebo‐Rd (17.5 vs. 11.5 months; *p* = 0.005), which translated to an improved median OS (36.6 vs. 26.0 months; *p* = 0.008) [24]. In this post hoc subgroup analysis of the TOURMALINE‐MM1 trial, a similar significant PFS benefit with ixazomib‐Rd over placebo‐Rd (18.7 vs. 10.2 months, HR, 0.504, *p* = 0.008) was observed.

In this study, the effect of confounding factors such as subsequent therapy with novel agents was minimised, revealing the potential treatment benefit of ixazomib‐Rd in patients with RRMM, especially among those with a poor prognosis. However, the study had some limitations. The main study was designed to have adequate power to detect benefit in the overall population but was not powered to detect subgroup effects. Owing to the inclusion criteria, the sample size was small and could have led to estimation bias or error in the analysis of survival advantage with ixazomib‐Rd treatment, especially when patients were further divided into smaller subgroups. Nevertheless, the current results provide context on the treatment of patients with RRMM in the emerging markets.

## 5 CONCLUSION

Overall, patients with RRMM from emerging markets showed a clinically meaningful benefit in survival with ixazomib‐Rd compared with placebo‐Rd, with no new toxicity or safety signals. Ixazomib‐Rd, an all‐oral triplet regimen, represents an effective and well‐tolerated treatment option for patients with RRMM, particularly in countries where novel treatment options are limited.

## AUTHOR CONTRIBUTIONS

Andrew Spencer contributed to data collection. Olga Samoilova contributed to data collection. Wee‐Joo Chng contributed to data analysis and interpretation. Richard Labotka has contributed to study design and data collection. Cong Li contributed to the statistical analysis of data. Kwang‐Wei Wu contributed to study design, concept development and analysis and interpretation of clinical data. Nakul Saxena contributed to data analysis and interpretation. Xu Yan supported with patient enrolment, treatment and evaluation. Jae Hoon Lee supported with patient enrolment. Meral Beksac contributed to data collection and analysis. All authors are accountable for the integrity of this work, provided critical revision of the manuscript and approved the final version for submission.

## CONFLICT OF INTEREST

Nakul Saxena is an employee of Takeda Pharmaceuticals International AG, Singapore and holds stocks of Takeda Pharmaceuticals. Kwang‐Wei Wu was an employee of Takeda Pharmaceuticals International AG, Singapore, during the conduct of this study and holds stocks of Takeda Pharmaceuticals. Her present affiliation is Janssen Pharmaceuticals (as of February 2022). Richard Labotka and Cong Li are employees of Takeda Development Center America and hold stocks of Takeda Pharmaceuticals. Meral Beksac has received honoraria from Amgen, Janssen, Sanofi, Oncopeptides, Celgene and Takeda. Andrew Spencer, Olga Samoilova, Wee‐Joo Chng, Xu Yan and Jae Hoon Lee state no conflict of interest.

## DATA SHARING STATEMENT

The datasets, including the redacted study protocol, redacted statistical analysis plan and individual participants data supporting the results reported in this article, will be made available within three months from initial request, to researchers who provide a methodologically sound proposal. The data will be provided after de‐identification, in compliance with applicable privacy laws, data protection and requirements for consent and anonymisation.

## ETHICS STATEMENT

The study was conducted in accordance with the International Conference on Harmonisation Good Clinical Practice guidelines and appropriate regulatory requirements. The study was approved by the local ethics committees or institutional review boards at each centre. All patients provided written informed consent.

## Supporting information

Supplementary data
