# Supplementary material for: Impact of ixazomib‐lenalidomide‐dexamethasone therapy on overall survival in multiple myeloma patients: Analysis of the emerging‐markets subgroup of the TOURMALINE‐MM1 trial
Source: EJHaem. 2022 Aug 31;3(4):1241–51. doi: 10.1002/jha2.548 (PMC9713050; doi:10.1002/jha2.548)
Supplement: Supplementary file 1 — Supplementary data [file JHA2-3-1241-s001.docx]

**Supplementary data**

**TABLE S1** Extent of exposure and study drug compliance

| Category | Ixazomib-Rd  n=70 | Placebo-Rd  n=77 |
| --- | --- | --- |
| Number of treated cycles^a^ |  |  |
| Mean (SD) | 12.9 (6.16) | 11.2 (5.57) |
| Median (min, max) | 13.0 (1, 26) | 12.0 (1, 25) |
| Number of treated cycles, n (%) |  |  |
| ≥1 | 70 (100) | 77 (100) |
| ≥2 | 68 (97) | 73 (95) |
| ≥3 | 67 (96) | 70 (91) |
| ≥4 | 65 (93) | 68 (88) |
| ≥5 | 61 (87) | 66 (86) |
| ≥6 | 60 (86) | 65 (84) |
| ≥7 | 58 (83) | 61 (79) |
| ≥8 | 55 (79) | 58 (75) |
| ≥9 | 52 (74) | 52 (68) |
| ≥10 | 46 (66) | 50 (65) |
| ≥11 | 46 (66) | 46 (60) |
| ≥12 | 45 (64) | 41 (53) |
| ≥13 | 38 (54) | 31 (40) |
| ≥14 | 34 (49) | 24 (31) |
| ≥15 | 26 (37) | 19 (25) |
| ≥16 | 22 (31) | 13 (17) |
| ≥17 | 20 (29) | 12 (16) |
| ≥18 | 17 (24) | 12 (16) |
| ≥19 | 17 (24) | 10 (13) |
| ≥20 | 12 (17) | 6 (8) |
| ≥21 | 7 (10) | 3 (4) |
| ≥22 | 5 (7) | 2 (3) |
| ≥23 | 4 (6) | 2 (3) |
| ≥24 | 3 (4) | 1 (1) |
| ≥25 | 1 (1) | 1 (1) |
| ≥26 | 1 (1) | 0 |
| Extent of exposure (cycles)^b^, n (%) |  |  |
| 1–3 | 5 (7) | 9 (12) |
| 4–6 | 7 (10) | 7 (9) |
| 7–9 | 12 (17) | 11 (14) |
| 10–12 | 8 (11) | 19 (25) |
| 13–15 | 16 (23) | 18 (23) |
| 16–18 | 5 (7) | 3 (4) |
| ≥19 | 17 (24) | 10 (13) |
| Extent of exposure (days)^c^ |  |  |
| n | 70 | 77 |
| Mean (SD) | 358.0 (178.09) | 311.0 (158.54) |
| Median (min, max) | 361.0 (19, 707) | 327.0 (7, 690) |

max, maximum; min, minimum; Rd, lenalidomide and dexamethasone; SD, standard deviation.

^a^A treated cycle is defined as a cycle in which the patient received any amount of ixazomib-Rd or placebo-Rd.

^b^Extent of exposure (cycles) is based on the number of treated cycles.

^c^Extent of exposure (days) is calculated as last dose date – first dose date + 1.

**TABLE S2** Common AEs in the safety population

| TEAE, n (%) | Ixazomib-Rd^a^  n=70 | | Placebo-Rd^b^  n=77 | |
| --- | --- | --- | --- | --- |
|  | Any grade | Grade ≥3 | Any grade | Grade ≥3 |
| Diarrhoea | 37 (52.9) | 7 (10.0) | 29 (37.7) | 0 (0.0) |
| Upper respiratory tract infection | 28 (40.0) | 1 (1.4) | 28 (36.4) | 1 (1.3) |
| Nausea | 27 (38.6) | 1 (1.4) | 20 (26.0) | 0 (0.0) |
| Fatigue | 24 (34.3) | 2 (2.9) | 24 (31.2) | 4 (5.2) |
| Constipation | 23 (32.9) | 0 (0.0) | 19 (24.7) | 0 (0.0) |
| Peripheral neuropathy^d^ | 23 (32.9) | 1 (1.4) | 17 (22.1) | 0 (0.0) |
| Vomiting | 21 (30.0) | 1 (1.4) | 17 (22.1) | 1 (1.3) |
| Back pain | 21 (30.0) | 0 (0.0) | 19 (24.7) | 5 (6.5) |
| Insomnia | 20 (28.6) | 5 (7.1) | 20 (26.0) | 0 (0.0) |
| Peripheral oedema | 17 (24.3) | 1 (1.4) | 17 (22.1) | 0 (0.0) |
| Neutropenia | 16 (22.9) | 13 (18.6) | 16 (20.8) | 12 (15.6) |
| Anaemia | 16 (22.9) | 7 (10.0) | 27 (35.1) | 18 (23.4) |
| Rash^d^ | 16 (22.9) | 2 (2.9) | 14 (18.2) | 2 (2.6) |
| Muscle spasms | 16 (22.9) | 0 (0.0) | 19 (24.7) | 0 (0.0) |
| Thrombocytopenia^d^ | 13 (18.6) | 9 (12.9) | 12 (15.6) | 6 (7.8) |
| Pneumonia | 13 (18.6) | 7 (10.0) | 14 (18.2) | 11 (14.3) |
| Nasopharyngitis | 11 (15.7) | 0 (0.0) | 8 (10.4) | 0 (0.0) |
| Cataract | 11 (15.7) | 2 (2.9) | 11 (14.3) | 5 (6.5) |
| Hypotension | 6 (8.6) | 1 (1.4) | 1 (1.3) | 0 (0.0) |
| Liver impairment^d^ | 5 (7.1) | 1 (1.4) | 6 (7.8) | 2 (2.6) |
| Heart failure^c d^ | 1 (1.4) | 1 (1.4) | 4 (5.2) | 2 (2.6) |
| New primary malignant tumour^e^ | 12 (16.9) | | 10 (13.0) | |

AE, adverse event; ITT, intent-to-treat; MedDRA, Medical Dictionary for Regulatory Activities; Rd, lenalidomide and dexamethasone; TEAE, treatment-emergent adverse event.

^a^12 and 5 patients in the ixazomib-Rd group had grade 4 and 5 AEs, respectively.

^b^16 and 8 patients in the placebo-Rd group had grade 4 and 5 AEs, respectively.

^c^2 patients in the placebo-Rd group had grade 5 heart failure.

^d^Values are presented for High Level Term as per MedDRA classification. Unless specified, all other AEs are presented with values for preferred term.

^e^This analysis was conducted in the ITT population. In the ixazomib-Rd group, the incidence of malignancies was as follows: non-haematological cancer (50%), non-melanoma skin cancer (50%), melanoma (8%) and haematological cancer (0%). Incidence in the placebo-Rd group was 30%, 70%, 0% and 10%, respectively. The percentages are based on the number of patients with new primary malignancy in each group.

**FIGURE S1** HRQoL scores from (A) EORTC QLQ-C30^a^, (B) and (C) EORTC QLQ-MY20^a^ over time^b^

**
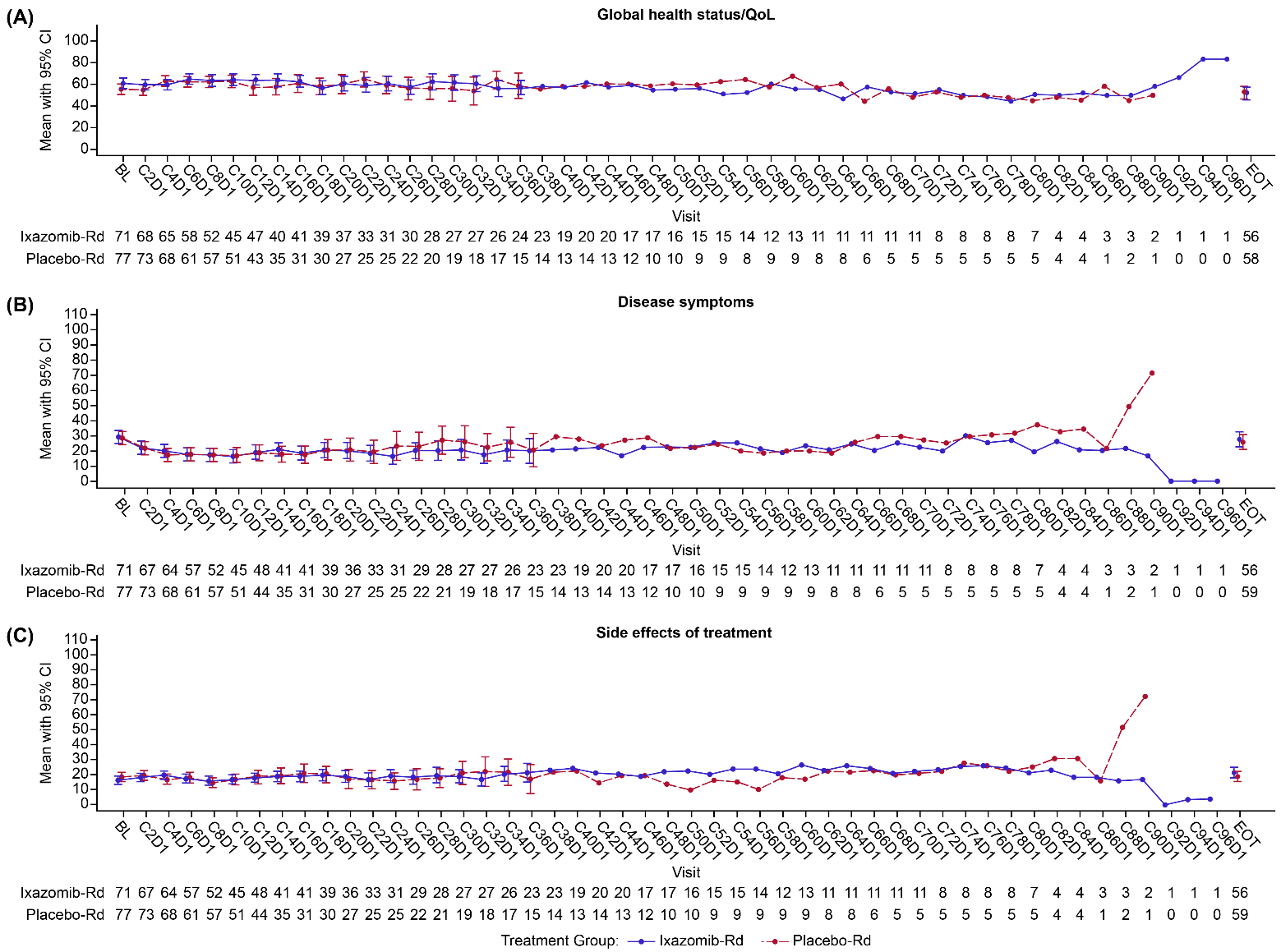
**

BL, baseline; CI, confidence interval; EORTC QLQ-C30, European Organisation for Research and Treatment of Cancer Quality of Life Questionnaire Core 30; EORTC QLQ-MY20, European Organisation for Research and Treatment of Cancer Quality of Life Questionnaire Multiple Myeloma Module 20; EOT, end of treatment; HRQoL, health-related quality of life; Rd, lenalidomide and dexamethasone; QoL, quality of life.

^a^Scores are linearly transformed to a 0–100 scale. Confidence bounds less than 0 and greater than 100 are truncated at 0 and 100, respectively. High scores for the global health status/QoL and functional domains indicate higher quality of life or functioning. Higher scores on the symptom scales represent higher levels of symptomatology or problems. Confidence bounds are suppressed if there are fewer than 15 subjects in either treatment arm.

**^b^**The timeframe is from BL to cycle X (CXD1) or EOT, whichever comes earlier. The QoL scores were collected every other cycle, after cycle 2 (C2D1), ie, cycles 4, 6, 8 and so on during the treatment period.
